# Biologging of emperor penguins—Attachment techniques and associated deployment performance

**DOI:** 10.1371/journal.pone.0265849

**Published:** 2022-08-04

**Authors:** Aymeric Houstin, Daniel P. Zitterbart, Alexander Winterl, Sebastian Richter, Víctor Planas-Bielsa, Damien Chevallier, André Ancel, Jérôme Fournier, Ben Fabry, Céline Le Bohec

**Affiliations:** 1 Département de Biologie Polaire, Centre Scientifique de Monaco, Monaco, Principality of Monaco; 2 CNRS UMR 7178, IPHC, Université de Strasbourg, Strasbourg, France; 3 Department of Physics, Friedrich-Alexander-University Erlangen-Nürnberg, Erlangen, Germany; 4 Applied Ocean Physics and Engineering Woods Hole, Woods Hole Oceanographic Institution, Woods Hole, MA, United States of America; 5 International Research Organization for Advanced Science and Technology (IROAST), Kumamoto University, Kumamoto, Japan; 6 CNRS UMR 7204 CESCO, Station de Biologie Marine, Muséum National d’Histoire Naturelle, Concarneau, France; 7 Centre de Recherches sur la Biologie des Populations d’Oiseaux, Muséum National d’Histoire Naturelle, Paris, France; MARE – Marine and Environmental Sciences Centre, PORTUGAL

## Abstract

An increasing number of marine animals are equipped with biologgers, to study their physiology, behaviour and ecology, often for conservation purposes. To minimise the impacts of biologgers on the animals’ welfare, the *Refinement* principle from the Three Rs framework (*Replacement*, *Reduction*, *Refinement*) urges to continuously test and evaluate new and updated biologging protocols. Here, we propose alternative and promising techniques for emperor penguin (*Aptenodytes forsteri*) capture and on-site logger deployment that aim to mitigate the potential negative impacts of logger deployment on these birds. We equipped adult emperor penguins for short-term (GPS, Time-Depth Recorder (TDR)) and long-term (*i*.*e*. planned for one year) deployments (ARGOS platforms, TDR), as well as juvenile emperor penguins for long-term deployments (ARGOS platforms) in the Weddell Sea area where they had not yet been studied. We describe and qualitatively evaluate our protocols for the attachment of biologgers on-site at the colony, the capture of the animals and the recovery of the devices after deployment. We report unprecedented recaptures of long-term equipped adult emperor penguins (50% of equipped individuals recaptured after 290 days). Our data demonstrate that the traditional technique of long-term attachment by gluing the biologgers directly to the back feathers causes excessive feather breakage and the loss of the devices after a few months. We therefore propose an alternative method of attachment for back-mounted devices. This technique led to successful year-round deployments on 37.5% of the equipped juveniles. Finally, we also disclose the first deployments of leg-bracelet mounted TDRs on emperor penguins. Our findings highlight the importance of monitoring potential impacts of biologger deployments on the animals and the need to continue to improve methods to minimize disturbance and enhance performance and results.

## Introduction

In recent decades, biologging technology—the “use of miniaturised animal-equipped tags for logging and/or relaying data about an animal’s movements, behaviour, physiology and/or environment” [1]—has rapidly progressed and led to fundamental advances in ecology of *e*.*g*. terrestrial [2] and marine predators [3, 4] including seabirds [5–8]. This technical evolution that included miniaturisation, design optimisation, storage capacity and power consumption, was supported by the development of new analytical techniques and processing software [9, 10].

Biologgers can cause discomfort to the tagged animal and may even impede their movements, especially in the case of diving seabirds like penguins where the increased water drag can increase the energy expenditure [11–13]. However, the miniaturisation of devices [14], the establishment of guidelines [15, 16] and the activities of study review boards that oversee the ethical treatment of animals in scientific studies [17–19] help to mitigate negative impacts and to comply with the Three Rs framework (*Replacement*, *Reduction*, *Refinement*; [20]). Nonetheless, data obtained from biologgers are often of such importance for conservation biology that the benefits may outweigh the risk for the animals [21, 22], if the risks to animals are kept minimal. For example, tracking studies that determine the home range and movement corridors of species are often a prerequisite for conservation management policies [22–24]. If animal sensing data may be used for virtuous causes of scientific knowledge production or environmental advocacy [25–27], it may also serve as a way of filling data gaps to advance the interests of state or corporate industries, and not necessarily to reduce biological or environmental harm [28].

Yet, especially in the case of penguin tracking studies, the inability to observe the animals carrying the devices at sea bears the risk that deleterious effects may not be obvious [13] or may even remain unnoticed if birds are not resighted. For instance, after decades of flipper banding thousands of penguins [29]; and references in Jackson and Wilson [30], it was only in the 2000’s that studies [31–33] assessed its long-term effect, and showed that flipper bands dramatically decreased the survival and breeding success of their carrier. This finding raised important questions about ethics and bias in scientific studies. Flipper banding of penguins is a prime example of why it is necessary to study potential impacts of device deployments on animals.

To ensure data is of exemplary quality from a scientific and ethical point of view, the potential deleterious effects of deployment procedures (capture-attachment-recapture) must be assessed and mitigated. For instance, of the six studies [34–39] where emperor penguins (*Aptenodytes forsteri*) have been tagged with external biologgers for a year-round deployment duration, none has reported the recovery of the device or a sighting of an equipped bird after deployment. The causes of signal loss remained unclear [40, 41] and the fate of the device-carrying birds uncertain. The technique used by these studies was to glue the device directly to the birds’ feathers, a method that ensured supposedly for longer deployment duration. While never shown in emperor penguins, Wilson and colleagues [38] raised potential concerns regarding feather damage induced by this technique.

In this study, we present highly detailed procedures to capture, recapture, and externally attach telemetry devices on-site on emperor penguins. Different biologger types were chosen and deployed to answer multiple research questions while being subject to seasonal constraints. We document for the first time the resighting and recapture of long-term equipped emperor penguins as well as device retrieval and thus reveal the impact of using glue for biologging device attachment on emperor penguins while proposing alternatives. We present the first leg-band biologger attachment and deployment on emperor penguins. Leg-band biologger attachment and deployment has also been used with TDR or global location sensor (GLS) on various penguin species, including Adélie, rockhopper (*Eudyptes chrysocome*), gentoo (*Pygoscelis papua*), macaroni (*Eudyptes chrysolophus*) and magellanic (*Spheniscus magellanicus*) penguins causing little behavioural disturbance and not jeopardizing birds’ survival [42–45]. To date, no such deployment had been reported on emperor penguins. Additionally, we describe and discuss methods for catching, handling or retrieving (resight and recapture) emperor penguins. These necessary procedures lack standardisation across studies. Some use a rugby-like catch method [46, 47], others would use a crook [40, 48] or a fixed enclosure [49, 50], and the impacts of these procedures on the targeted bird are rarely reported.

Summarising, in this manuscript, we describe and review protocols for on-site capture, handling and release of emperor penguins as well as biologger attachment and recovery techniques that aims to minimise the impacts on the birds’ welfare.

## Methods

### Ethics statement

The AWI long-term program “MARE” (Monitor the health of the Antarctic maRine ecosystems using the Emperor penguin as a sentinel), to which this study belongs, and all procedures were approved by the German Environment Agency (Umweltbundesamt-UBA permit no.: II 2.8–94033/100 delivered on the 04/10/2017 and 04/10/2018), and conducted in accordance with the Committee for Environmental Protection (CEP) guidelines.

### Study site

This study was conducted at the Atka Bay emperor penguin colony (70°37’S, 08°09’W) in close vicinity (~ 10 km) of the German research base Neumayer Station III (70°39’S, 08°15’W) during two consecutive summer campaigns (November to January 2017–2019). During these campaigns, we deployed biologgers (see S1 Table in S1 File) for short-term, i.e. few weeks in summer, and long-term duration, i.e. year-round planned monitoring that include austral winter.

### Study species

The deployment protocols possible to implement on emperor penguins largely depend on the species’ phenology (and logistic constraints). The Emperor penguin is the only bird species breeding during the austral winter [51], almost exclusively on sea ice [52] all around Antarctica [53]. After a courtship period in March and April, depending on the colony’s latitude, and an incubation period of around 64 days, the chicks hatch in the middle of the austral winter. As central place foragers [54], male and female alternate trips at sea to find food for their sole offspring. By October, the chick is thermally independent and is left on its own while both parents go foraging at sea and return to feed their chick independently [51, 55]. These recurring returns of each adult to feed its chick, approximately once per week [51, 55], allow deployment and retrieval of short-term data loggers. In December or January, chicks moult and fledge. By the end of the austral summer, the adult emperor penguins moult. For both, moulted chicks (*i*.*e*. juveniles) and adults, a reliable attachment of long-term logging devices on their back is only possible after moulting is largely completed. The majority of juvenile birds will not return to the colony for at least two years [56] and previous studies suggest that most of the adults moult on the pack ice [36, 57–59]. There is also no certainty that the very few adults moulting at the colony are actual breeders from that particular colony and that they will return in the next season. Therefore, successfully retrieving the devices is unlikely and the use of transmitting devices is by far the most prevalent technique to ensure data collection.

### Capture methods

A very limited number of scientists have ever handled a non-anaesthetized adult emperor penguin. Handling such an animal can be difficult as they are strong but fragile birds (especially the flippers) with a body mass ranging from 15 (this study) to *ca*. 40 kg depending on age, sex, season and location [51, 60]. While it is always better to transfer such skills directly in the field, this may not always be possible due to the limited number of qualified and experienced persons able to train others. Therefore, our study and the associated protocols aim to fill part of this gap. The techniques developed in this study to approach, capture and handle an adult emperor penguin require, as a minimum, two qualified field staff (referred to hereafter as handlers).

#### Adult-chick capture protocol

Here, we present a technique to capture an adult emperor penguin with its thermally independent chick during the late chick-rearing period. To avoid larger disturbances, it is ideal to capture birds at the outer edge of the colony. Therefore, the first step is to observe the outermost 3–4 rows of animals from a distance, to locate adults that are feeding the same chick several times in 30–60 minutes and that are either stationary or moving towards the outer edge of the colony. Note that allofeeding behaviour is quite common in emperor penguins [61] but allofeeders usually do not stay with the same chick at this time of the season (Houstin and Le Bohec, unpublished observations).

**Fig 1:**
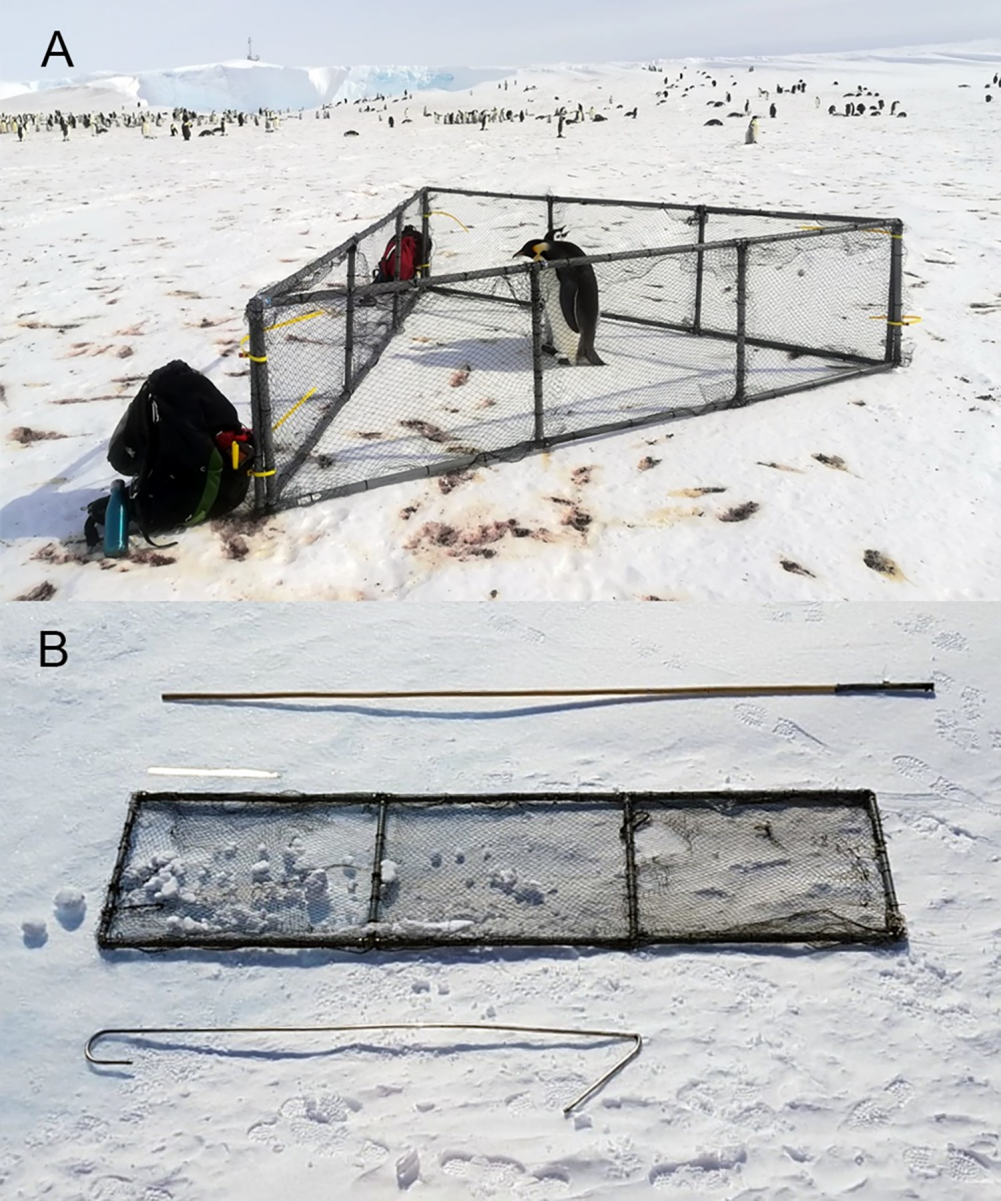
Required tools to capture emperor penguins. **(A)** An adult-chick pair inside the corral. **(B)** A 3 m long bamboo stick at the top, one of the panel of the corral (a 50 cm ruler is placed just above it to facilitate scaling) in the middle, and the crook at the bottom. The crook is the right extremity of the pole while the left extremity is a hook.

*Capture equipment*. Three main tools are required:

- One 2 to 3 m long stick (*e*.*g*. lightweight bamboo sticks) used to direct targeted birds out of the colony (Fig 1A).
- One 2.5 m long light crook, designed to go around the penguin’s neck, made of stainless steel or aluminium, bent at 50 cm from one end by an angle of approximately 135° (Fig 1A), used to direct birds and catch them if necessary. Note that a crook is more efficient than a hook. The wide opening of a crook, combined with its narrow angle in comparison to a hook, reduces the ability of penguins to escape by twisting their neck.
- A corral made of three separate panels (Fig 1A). Each panel consists of plastic pipes joined together to form a 3 m by 0.8 m frame. For every meter in length, a vertical plastic tube is added for stability. A polypropylene net (aviary net with a mesh size of 2 cm) is connected to the frame using cable ties. This construction results in lightweight (*e*.*g*. 4.5 kg), sturdy and field serviceable panels. When the panels are connected (Fig 1B), the triangle formed can be closed with two large reusable cable ties at each of the three joints. We suggest covering one of the panels with a plain fabric, even if this makes the panel more difficult to handle when there is wind. The fabric reinforces the corral, provides shade to the birds and prevents them from attempting to go through the net. It also protects the fieldworkers from wind and allows them to hide behind the panel to calm the birds before release.

*Corral capture procedure*. When the target adult-chick pair is located, the two handlers (one with the bamboo stick, and the other one with the crook) move towards the birds from two sides, starting approximately 40 m away from the colony. The first step for the handlers is to position themselves “behind” the pair, so that the birds are between them and the outer edge of the colony. The second step is to guide the pair slowly out of the colony by walking one-step at a time behind them. The handlers move very slowly to minimise the disturbance of the colony. The resulting disturbance is minimal (S1 Movie in S1 File) especially if compared to a natural event like the intrusion of a Weddell seal (*Leptonychotes weddellii*) into the colony (S2 Movie in S1 File). Meanwhile, the two assistants are positioned at a distance of approximately 100 m from the colony edge with the three corral panels and await instructions by radio communication at a minimal volume. Situational awareness is crucial to anticipate the location where the pair will exit the colony and to ensure the least possible disturbance during capture.

Once the pair is ~30–40 m outside the colony, one assistant hands one of the panels to the handler with the bamboo stick, and returns to his own position. Once the panels have been placed equidistantly (~30 m) around the penguin pair, everybody moves closer to the pair and closes the corral around it. It is to be noted that the last few meters (< 5 m) before the corral is fully closed, the team has to move in a smooth, swift and highly coordinated manner, so that no escape route is presented. If correctly executed, the penguins will remain stationary, looking for the best escape route, and find themselves in the closed corral before an escape is attempted. The handler with the crook helps to close and secure the corral with reusable cable ties. If the adult attempts to escape, use the crook to catch the bird and prevent the escape (see section below—single adult capture protocol). Four persons are the optimal number to carry out this capture protocol. If everybody is experienced, it can be executed comfortably, for the animals as well as for the scientists, with three people. The whole procedure is presented in detail in S1 Movie in S1 File. After capture and manipulation, we recommend letting parent and chick rest and calm down for a few minutes in the corral to increase the chance that they stay together upon release. To release the birds in a particular direction, the cable ties of the edge facing the desired exit direction are unzipped and the corral sufficiently opened to allow the birds to go through (S3 Movie in S1 File).

We used this method to capture a pair of one adult with its not-yet-moulted chick to increase our chances of recapture. Indeed, breeders with moulted chicks ready to fledge or with chicks having sufficient reserves to perform their moult and fledge on their own are more prone to end their breeding cycle, defined by the “abandonment” of their chick [46]. Devices were recovered after one to three foraging trips.

#### Single adult capture protocol

Two techniques can be used to capture a single adult emperor penguin; the choice depends on the behaviour of the bird while approaching, the availability of assistants, and the weather conditions.

As described above for the pair of an adult and its chick, the corral can be used to trap a single adult in a very similar way. Nonetheless, due to the fact that solitary birds are more mobile and usually more vigilant to their environment the corral method may be difficult, which is especially true during heavy winds.

An alternative and efficient technique is to use a crook to catch the bird (S4 Movie in S1 File) as explained in [48]. The crook capture requires two people and in contrast to the corral protocol and the deployment of loggers can also be conducted in bad weather. Once the bird is isolated, one handler places the crook around the neck of the bird preventing the penguin from escaping by tobogganing, *i*.*e*. moving on its belly. Meanwhile, the other handler grabs the tibiotarsi of the bird and holds them firm. The crook is gently removed and placed away from the capture site, and the penguin secured by the two handlers, one in front of the bird and one at the back. The crook-carrying handler should be carrying the necessary supplies for manipulation in a backpack, because, after the capture, the crook-carrying handler will have free hands, while the other handler is still holding the bird.

We used this technique to recapture adults for device recovery or to capture non-breeding (*e*.*g*. moulting or post-moulting) adults.

#### Fledging juvenile capture protocol

For their first departure at sea, juvenile emperor penguins usually leave the colony in small groups. A group capture with the corral is, therefore, more efficient and potentially less stressful for the birds. The protocol is similar to the adult-chick-pair capture, but here an entire group of juveniles is slowly encircled by three corral bearers. As emperor penguins are social animals, it is likely that keeping the group together reduces the stress of manipulated individuals and facilitates the remainder of their travel towards the sea after release. Juveniles of interest are removed individually from the corral for the manipulation and returned afterwards. All juveniles are released together after all target animals have been handled.

### Adult emperor penguin handling protocol

Similarly to [48], and as shown in the S5 Movie in S1 File, the bird is caught and kept in an upright position by one handler (H1). Once the bird is secured in this position, the second handler (H2) approaches and bends the penguin’s head towards the ground while H1 grabs the legs above the ankles to lay the bird on its belly. When the bird is lying on the ground, H2 kneels over the bird with its head between (below) the legs of H2. In this position, the bird is immobilised. It is crucial that the flippers, the most fragile part of the bird, are unrestrained and untouched, throughout the whole process. An available assistant, if any, can hold the legs of the birds and stretch them (foot soles pointing towards the sky). Working with three people allows H1 to deploy the loggers seated next to the penguin and reduces manipulation time. Most penguins stay quiet in this position with some few second long bursts of intense activity: a gentle but firm pressure on the back and pulling the foot soles upper and further from the ground helps to calm the bird.

### Instrumentation

We caught three categories of birds (a pair of one adult with its chick in November/December, juveniles and moulted adults in January) in order to deploy and/or recover six different types of loggers (S1 Table in S1 File). Depending on the duration (short- or long-term) of planned equipment, biologgers were attached by one of four techniques: back-attachment-tape/-cyanoacrylate-glue/-tape-epoxy and leg-band (Table 1). Additionally, all birds, *i*.*e*. adults and chicks, were marked with subcutaneous passive integrated transponder (PIT of 3.85 × 32 mm and 0.8 g, Texas Instruments Remote Identification System, TIRIS, Texas, USA) implanted between the tail and left leg (S6 Movie in S1 File) allowing remote identification of individuals with automatic reading systems, *i*.*e*. not requiring the recapture of birds. The size of the PIT allows for a current detection distance by the systems of approximately 60 cm. All protocols adhered to current best-practise standards to reduce the risk of physical harm and stress to individuals and the colony. During manipulation, the bird’s eyes were always covered with a hood to reduce stress level [18] and birds were handled at distance from the edge of the colony to avoid conspecifics’ disturbance (usually > 40 m, thus well above the 5 m limit recommended in the General guidelines produced by the Antarctic Treaty Consultative Meeting [62].

**Table 1:** General information on deployments and captures.

Category of attachment	Back-taped	Leg-banded	Back-glued	Back-taped-epoxied
**Deployment duration**	Short-term	Long-term		Long-term
**Age class**	ADULT	ADULT		JUVENILE
**Logger type**	GPS—VHF - TDR	TDR—ARGOS		ARGOS
**Monitored period**	summer	winter		winter
**Number of individuals equipped**	36	8		8
**Average mass of birds (mean** ± **sd in kg)**	27.3 ± 2.7	20.83 ± 2.6		14.00 ± 1.32
**Capture setup**	with its chick at the colony edge	alone at the edge of groups		groups on their way to the sea
**Capture technique**	corral + crook	crook or corral		corral
**Minimum persons required**	3	2 to 3		3
**Recapture technique**	crook	crook		not possible, not returning
**Minimum persons required for recapture**	2	2		to breeding site before moult

### Instrument attachment methodology and deployments

#### Short-term deployment on adults: Back-taped loggers

Before starting with the attachment, we used a cardboard stencil and waterproof tape that is a bit larger than the logger to demarcate the precise location of the equipment and the placement of the strips of adhesive tape on the penguin (see this in detail in S7 Movie in S1 File). Following previous studies that used the tape technique for short-term deployments [59, 63–68], we used a rounded knife to lift a few feathers from the back of the penguin and insert pre-cut strips of waterproof adhesive tape (*e*.*g*. Tesa® tape 4651, Beiersdorf AG, Hamburg, Germany). To further reinforce the attachment, we added glue (*e*.*g*. cyanoacrylate glue, Loctite 401, Loctite, Henkel AG., Düsseldorf, Germany) between the adhesive part of the tape strips and the logger. Cable ties (*e*.*g*. Panduit, Panduit Corp, Illinois, USA) should be tightened with a cable tie gun. For a deployment period of more than one month, we recommend to add glue on top of the tapes. After manipulation was completed, we marked the bird before release with a hair-dye painted number that will last until the following moult (*e*.*g*. Schwarzkopf, Palette dark-blue N°909, Henkel AG., Düsseldorf, Germany, see S1 Fig in S1 File to behold a marked bird).

**Fig 2:**
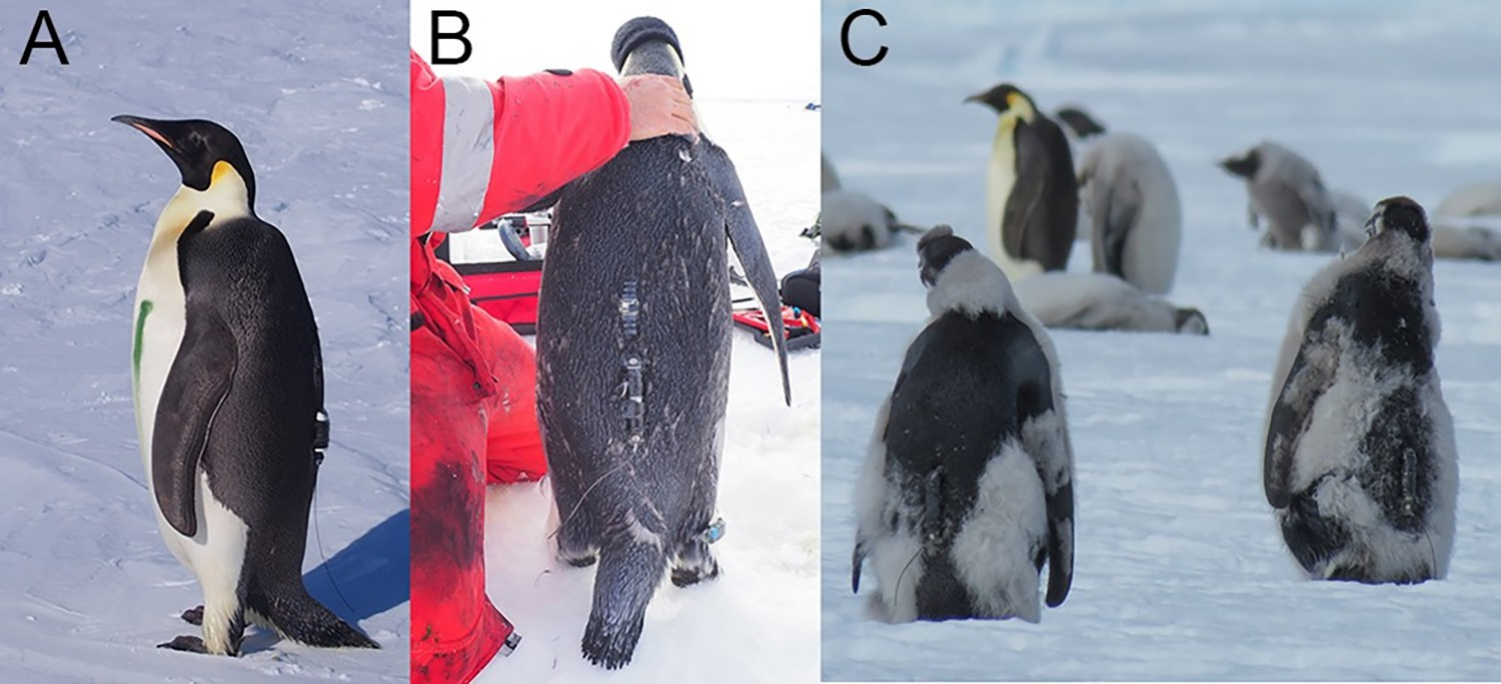
Pictures of the different deployments performed. **(A)** Adult emperor penguin equipped with back-taped loggers (a TDR in the middle of the back and a GPS underneath). The green line on the bird’s belly is non-permanent marking. **(B)** Adult emperor penguin equipped with back-glued loggers (an accelerometer in the middle of the back and ARGOS satellite transmitter underneath) and a leg-banded logger on its right foot. **(C)** Juvenile emperor penguins both equipped with a back-taped-epoxied logger.

We used this technique to equip 16 adults in 2017/2018 and 20 adults in 2018/2019 with a GPS-Acc-VHF logger (a combination of a Global Positioning System (GPS), a 3-axis Accelerometer (Acc) and a Very High Frequency (VHF) locator beacon; Axy-Trek, TechnoSmArt, Italy) and a separate Time-Depth Recorder (TDR; g5+, CEFAS, UK). Respective movies of deployments are presented in S7 and S8 Movies in S1 File and a fully equipped bird is shown in Fig 2A. Both loggers are archival devices and, therefore, need to be retrieved to download the data. The VHF locator beacon sends a device-specific signal that allows to locate the equipped birds in the colony and facilitate device recovery. To minimise deleterious effects such as extra drag on diving animals [11, 69], we followed the recommendations of previous studies. The devices represented less than 1% of the penguin’s cross-sectional area, weighed less than 3% of the bird’s mass (Table 1) [16] and were attached on the lower back of the birds.

#### Long-term deployment on adults: Back-glued loggers

Following attaching technique classically used by all the previous studies that conducted long-term deployment of biologgers on the back of emperor penguins, we affixed the lower side of the loggers directly to the feathers in the middle of the lower back of birds using cyanoacrylate glue (Loctite 401). In addition to the direct contact between the glue and the feathers, the attachment was secured with polyamide cable ties as described above (Fig 2B). Although Wilson and colleagues [38] raised potential concerns regarding feather damage induced by this technique in some penguin species, resulting impacts on emperor penguins have never been assessed despite the common use of this technique for long-term deployments on the species after this date [34–39].

In January 2018, using this technique, we equipped 8 adult emperor penguins close to finishing their moult (see S2 Fig in S1 File to identify an emperor penguin in that stage) with an Advanced Research and Global Observation Satellite (ARGOS) platform (Spot-367, Wildlife Computers, USA) and a separate accelerometer (WACU, MIBE-IPHC-CNRS, France). ARGOS platforms sent the birds’ location via the Collecte Localisation Satellites (CLS) ARGOS service (Toulouse, France).

#### Long-term deployment on juveniles: Back-taped-epoxied loggers

Similar to the short-term deployment protocol, the logger is attached to the feathers using pre-cut lengths of Tesa® tape on the entire logger length (sparing exposed sensors if any). The overlap between tape strips is reinforced with cyanoacrylate glue (Loctite 401). We used two polyamide cable ties around the head and one at the bottom of the logger to secure the attachment. The second cable tie on the head is added for extra safety. Once all the adhesive strips and cable ties were attached, we applied epoxy adhesive (Loctite EA 3430) on the mounting (sparing exposed sensors if any) to reinforce the waterproofness and robustness, adapting methods from other studies [70, 71]. The attachment procedure is shown in the S9 Movie in S1 File. Importantly, the epoxy glue did not come into contact with the back feathers.

In January 2019, using this technique, we equipped 8 fledging chicks with similar ARGOS devices (Spot-367, Wildlife Computers, USA) than the one presented for adults in the above section. We selected the individuals most advanced in their moult, *i*.*e*. presenting no more down on their back (Fig 2C). The lower survival rate of the juveniles during the first year at sea compared to adults [72], their non-return to breeding colonies before several years [56] and their unfinished growth, prevent the use of other types of externally attached devices.

#### Long-term deployment on adults: Leg-banded loggers

With the aim to reduce drag and behaviour disturbance induced by devices on the back of penguins, we developed a leg-band (bracelet) for mounting TDR-loggers on emperor penguins. Similar leg-bracelets had been successfully deployed on other penguin species [42–45, 73].

We designed two similar types of bracelet, a first version that we deployed (Fig 2B), and a second version incorporating slight changes and improvements. A detailed manual of the mounts is provided in S1 Slideshow in S1 File. We designed the bracelet to mount a Lotek Lat 1800 TDR (Lotek, Canada; S1 Table in S1 File) but the bracelet can be easily adapted to other types of TDR.

The TDR is fixed to a rubber cable tie (Panduit, ERTM-C20) covered with heat-shrinkable sheath and attached around the bird tibiotarsus by closing the cable tie just above the ankle, like a bracelet. A built-in lock prevents the cable tie to tighten itself after deployment. The bracelet fits loosely with ~1 cm space between the bracelet and the leg. The shape of emperor penguin’s legs prevents the bracelet from spinning around the leg (S1 Slideshow in S1 File). Deployment time lasts less than 3 minutes. On retrieval, the bracelet is easy and quick to remove (within a few seconds) by cutting the rubber cable tie with pliers.

In January 2018, the 8 birds equipped with ARGOS loggers (see section b above) were equipped simultaneously with an additional TDR sensor that was attached with a leg-bracelet.

## Results

### Short-term deployment on adults: Back-taped loggers

In 2017–2018, 16 deployments were performed: 10 between November 27^th^ and December 2^nd^, of which 6 devices were recovered and redeployed between December 10^th^ and 12^th^. None of the devices of the second deployment session were recovered, resulting in 38% recovery.

In 2018–2019, 20 deployments were performed, 10 between November 05^th^ and 07^th^, which all were recovered and redeployed between November 25^th^ and December 6^th^. Six devices of the second deployment were recovered, resulting in 83% recovery. We conducted intense VHF and visual (binocular) surveys for equipped birds (approx. every 4 hours), thus we are confident that we retrieved all the loggers from returning birds. All VHF units of recaptured birds were working and unequipped birds have been regularly identified afterwards by their hair-dye painted number on their chest.

Bird feathering on recovery was intact and no physical damage on the bird or on the device was apparent. All loggers were still securely attached, even after the longest deployment of 25 days (Table 2). Our recovery rate for November (90%) is similar (z-test, p-value > 0.05) to those of previous studies (Table 2). The recovery rate from December 2018 (30%), despite being higher, is statistically similar to what Robertson [46] recorded for deployments performed in December on the opposite side of Antarctica (near Australia’s Mawson Station) with a loss rate of 89%. The probability to recover a device deployed in December is significantly lower (z-test, p-value < 0.05) than in November.

**Table 2:** Comparison between at-sea-ecological studies that equipped emperor penguins over the last 30 years.

Category of deployment	Season	Age class	Type of device	Device dimension	Device weight	Rec-over	Mean duration	sd	Range	# deploy	% recup (#)	Publication	Study site and year
*long-term*	*W-Jan/Feb*	*ad*	*Argos*	*140x55x16*	*195*	*no*	*66*	*52*	*15–133*	*7*	*0 (0)*	*Kooyman et al*. *2004*	*Ross Sea 2000*
*long-term*	*W-Mar*	*ad*	*Argos*	*109x32x26*	*100*	*no*	*114*	*98*	*12–323*	*20*	*0 (0)*	*Goetz & Kooyman 2018*, *2015*	*Ross Sea 2013*
long-term	W-Jan	ad	Argos, acc & tdr	107x18x21 70x16x16 36x13x10	45 10 9	yes	150	30	118–201	8	50 (4)	This study	Atka Bay 2018
*long-term*	*W-Dec*	*juv*	*Argos*	*NA*	*120*	*no*	*64*	*12*	*43–81*	8*	*0 (0)*	*Kooyman et al*. *1996 and 2007*	*Cape Washington 1994*, *1995*, *1996*
*long-term*	*W-Dec*	*juv*	*Argos*	*NA*	*NA*	*no*	*113*	*49*	*41–160*	7*	*0 (0)*	*Wienecke et al*. *2010*	*Taylor glacier 1996*
*long-term*	*W-Dec*	*juv*	*Argos*	*NA*	*NA*	*no*	*121*	*55*	*38–189*	*10*	*0 (0)*	*Wienecke et al*. *2010*	*Auster 2007*
*long-term*	*W-Dec*	*juv*	*Argos*	*NA*	*62*	*no*	*112*	*77*	*38–255*	5*	*0 (0)*	*Thiebot et al*. *2013*	*Pointe Géologie 2010*
*long-term*	*W-Dec*	*juv*	*Argos*	*NA*	*62*	*no*	*193*	*93*	*30–344*	13*	*0 (0)*	*Labrousse et al*. *2019*	*Pointe Géologie 2014*
long-term	W-Jan	juv	Argos	107x18x21	45	no	233	108	73–382	8	*0 (0)*	This study	Atka Bay 2019
*short-term*	*S-Nov*	*ad*	*Argos & tdr*	*78x50x23*	*105*	*yes*	*7*	*1*	*2–19*	*15*	*67 (10)*	*Zimmer et al*. *2010*	*Pointe Géologie2005*
				*67x17x17*	*30*								
*short-term*	*S-Nov*	*ad*	*acc*	*128x27*	*101*	*yes*	*14*	*4*	*8–20*	*12*	*92 (11)*	*Watanabe et al*. *2012*	*Cape Washington 2005*
				*or 122x22*	*or 73*								
short-term	S-Nov	ad	gps & tdr	105x38x18	60	yes	16	6	9–25	21	90 (18)	This study	Atka Bay 2017 & 2018
				35x12	7								
*short-term*	*S-Dec*	*ad*	*tdr*	*NA*	*NA*	*yes*	*NA*	*NA*	*NA*	*19*	*11 (2)*	*Robertson*, *1991*	*Auster & Taylor glacier 1988*
short-term	S-Dec	ad	gps & tdr	105x38x18	60	yes	14	6	9–18	15	30 (4)	This study	Atka Bay 2018 & 2019
				35x12	7								

Comparison between at-sea-ecological studies that equipped emperor penguins over the last 30 years in terms of type of equipment, age class of birds and deployment duration. Only post-moult long-term deployments, *i*.*e*. pre-nuptial/pre-winter travels (beginning in January-March) and breeding short-term deployments, *i*.*e*. November-December, are considered in this table. Other studies have deployed devices specifically between May and October, *i*.*e*. breeding period only [8, 59, 74–77] or at the end of austral summer (end of December-January) trying to locate moulting areas [57, 58]. W-Mon = Winter-month of deployment, S = summer, ad = adult, juv = juvenile. Mean duration, sd and range are expressed in days. Device dimensions are expressed in mm and weight in g. NA = not available. * For studies on juveniles, duration below 30 days have been removed since those short period of deployment are mostly thought to be due to predation while for Kooyman et al. [39], only not hand-fed chicks have been considered. In bold, the greatest values within a category of deployment. In italic, data from other studies.

### Long-term deployment on adults: Back-glued loggers

**Fig 3:**
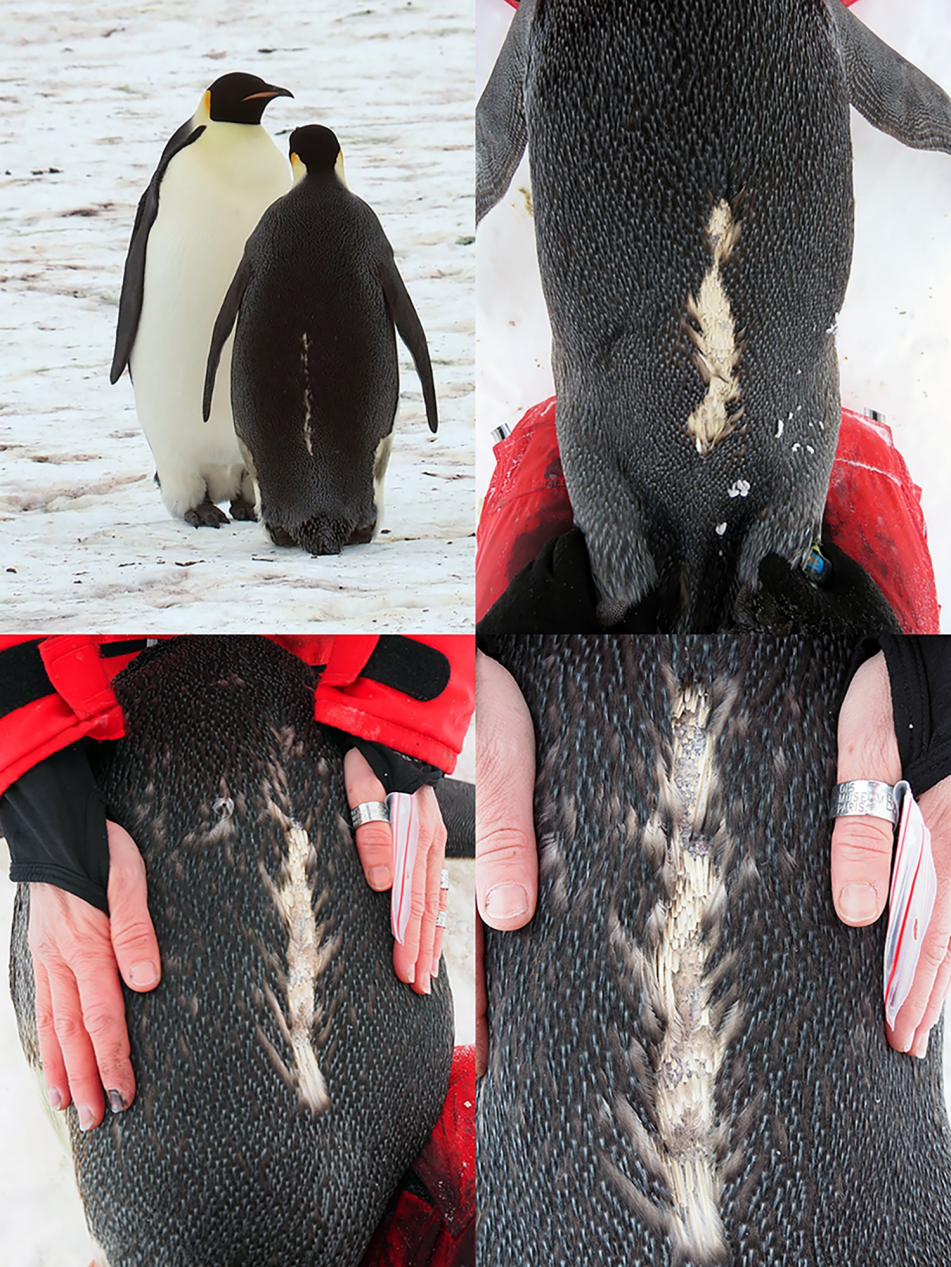
Back of the 4 penguins having lost their back-glued loggers during the winter.

Our study is the first to report recapture of emperor penguins after a whole-winter deployment (January to November). Identified by the number painted on their chest (S1 Fig in S1 File), 4 of the 8 birds equipped in January 2018 were resighted and recaptured in November 2018 (Table 2). All of them had lost the devices on their back. Instead, there was a line of missing/broken feathers (Fig 3). No injury was detected.

Signals from all ARGOS devices were lost during the winter. The mean transmission period was 150 ± 30 days (range 118–201 days, Table 2), significantly exceeding the previous average deployment durations of 66 [40] (p-value > 0.05, ANOVA) and 114 days [37] (p-value < 0.05, ANOVA) from all previous similar studies.

### Long-term deployment on juveniles: Back-taped-epoxied loggers

Three of the 8 juveniles equipped in January 2019 transmitted until their annual moult in January 2020. None of the birds did return to their native colony for moult; an observation congruent with the conjecture that juveniles of 1.5-years of age do not come back to moult at their birth colony for the first year [38, 56]. This tracking period of a full year, from January 2019 to January 2020 (Table 2) is the longest documented deployment duration for the genus Aptenodytes. None of the 5 remaining birds were spotted on colony despite visual search in summer 2019/2020. One device stopped transmitting after 73 days while the four others lasted between 142 and 185 days resulting in an average deployment duration of 233 ± 108 days. This mean deployment duration is longer than any previously reported (p-value > 0.05, ANOVA, see Table 2 for mean ± sd values) but not significantly compared to [38] (p-value < 0.05, ANOVA).

### Long-term deployment on adults: Leg-banded loggers

Our study is the first to perform a year-round deployment and data collection on emperor penguins. The 4 adults recaptured without their back-mounted loggers were still carrying their leg bracelet mounted TDR, providing an unprecedented record of an entire year of high frequency (1 Hz) depth and temperature logs for emperor penguins.

For all recaptured birds, the leg-bracelet mounting did not present any damage, the bracelet and the TDR were at the same position of their deployment, suggesting that the device did not rotate around the leg during the deployment period. However, all recaptured birds had lost a few feathers especially on the inside part of the leg and showed signs of abrasion in the form of a slight reddening of the skin and peeling under the bracelet area. Two of them had small spots of skin irritation on their tarsi. No limping was observed before or after removal. An illustrated comparison between an equipped and an unequipped leg after recovery can be found in the S2 Slideshow in S1 File.

## Discussion

To our knowledge, two of the four deployment methods presented in this study are new developments for this species and allowed for the longest documented deployment duration for this species. The description of those methods, paired with an exhaustive documentation, aims to facilitate and enhance future research on this species.

### Capture and handling

All capture techniques presented in this study yield minimal colony disturbance regardless of the period of the breeding cycle. The described handling is safe for birds and handlers, and only a minimal number of trained personnel is required. We recommend the use of the corral if no member in the field team is accustomed to handling a crook or a hook on at least one penguin species.

### Deployments

#### Short-term deployment: Back-taped loggers

Our study is the first to report the deployment of GPS devices on emperor penguins. The methods presented herein allow the deployment of these high-resolution data acquisition loggers with a high probability of recovery once the phenology of the colony has been assessed, for instance by the size and moulting stage of chicks.

At Atka Bay, the best deployment period is in November with a logger recovery rate of 90%. The low recovery rate (38%) during the 2017–2018 season can be explained by logistical issues we encountered and not the deployment technique. An unexpected late on-site arrival led to late deployment of 11 loggers in December 2017, compared to 4 in December 2018, and consequently to a substantial loss of devices.

To optimise the recovery rate of devices deployed at the end of the breeding season, we recommend deploying devices on adults with medium-sized chicks at the very beginning of chick moult. Supported by the secure attachment of the presented technique, we furthermore suggest increasing deployment time rather than to recover loggers and redeploy them.

#### Long-term deployment on adults: Back-glued loggers

Our study is the first to document the recapture of a long-term equipped emperor penguin and thus able to assess (i) the state of the bird, (ii) the state of devices, and (iii) provide for the first time ground-truth evidence explaining the loss of signal from communicating-satellite-relayed loggers for this species. The loss of the device inevitably leads to the loss of the signal. However, it is not possible to determine if the signal was potentially lost before the device fell off the bird. Until now, six studies [34–39] had performed long-term deployments on emperor penguins right after the moult (Table 2), all using ARGOS platforms directly glued to the back-feathers of the birds. None of the birds were resighted, perhaps partly due to the logistical difficulties to reach the colony in the following years at other study sites. Thus, only hypotheses can be made regarding the loss of signals in these studies (see particularly [40]). Devices may have remained attached on the birds until their moult while not transmitting anymore but our results combined with the use of the same attachment methods (simple gluing) strongly suggest otherwise. Our results show that both glued devices, the ARGOS transmitter and the small accelerometer, which vary in size and weight (S1 Table in S1 File), were lost by feathers’ removal for all birds.

We speculate that the cyanoacrylate glue rigidifies the feathers, which then become brittle and break with either the continuous birds’ movements and/or their attempts to remove the device. Wilson and colleagues [38] also observed this device-sized hole in the feather layer after winter deployment on four Magellanic penguins. Another possible explanation could be the timing of deployment. Devices were attached just at the end of the moult, a time when feathers may not yet be fully developed despite a meticulous bird selection process (S8 Movie in S1 File). Their growth after deployment could potentially have added some slack and thus reinforced the pull on the feather shafts, ultimately leading to their breakage after few months. The loss of back feathers undoubtedly leads to a diminution of insulation that causes a greater heat loss. The resulting increase in energetic needs reduces fasting capabilities and forces the birds to compensate by finding more prey items when they return at sea to forage in order to replenish their reserves while accumulating food for their chick. As body reserves management is critical, especially for this species, any significant heat loss is likely to impact breeding success. However, the fact that the mean weight of the four birds was on average 6.1 kg (range 2.2–11.5 kg) higher than at the time of their equipment suggests that their survival was not impaired.

In tagging procedures, the ethical principle of *Refinement* from the Three Rs [20], *i*.*e*. the use of methods which decrease any adverse effect, should apply. We consider the loss of devices and resulting consequences for the birds sufficient evidence, combined with the ones from [70], to recommend discontinuing the use of glue directly on the birds feathers for long-term deployment on penguins as it is currently practiced. We propose an alternative technique that has not yet been shown to induce such damage. The tape does not include acrylamide glue and is therefore less likely to brittle the feathers and therefore we expect the deployed device to remain on the back of the birds.

#### Long-term deployment on juveniles: Back-taped-epoxied loggers

So far, this age-class had only been tagged using the back-glued method (Table 2). Three of the juveniles (40%) retained their device for an entire year, thus achieving the longest duration of back-mounted logger deployment possible in penguins. The previous longest durations recorded for juvenile emperor penguins were of 344, 298 and 271 days [38] with one bird (6% of the deployments) approaching the one year length duration. The mean duration of our long-term deployments (233 ± 108 days) with the taped-epoxied technique is longer than any previously reported on juvenile or adult emperor penguins (Table 2). Therefore, we are confident that the technique presented is a significant improvement for tracking of penguins and understanding their activities at sea, even if the contribution of a possible gain resulting from the evolution of technologies is not measurable.

The lack of recovery of ARGOS devices deployed on juveniles can be explained by the fact that the birds moult outside their original colony [36, 57–59], or by the loss of devices as suggested by our results on adults. Electronic failure, bird predations or starvation are also alternative hypotheses [40, 78]. Nevertheless, in addition to the possible loss of feathers and insulation previously discussed, the glue has the potential to cause thermal skin burns [79]. Juveniles are more vulnerable than adults as their foraging skills (including their ability to dive, to capture prey, and to find productive feeding grounds) are not yet fully developed, and their experience to escape predators is also minimal [78, 80, 81]. The additional cost mentioned above induced by a glued device may negatively impact the survival of the juveniles during their first months in their new marine environment that they experience for the first time.

As a result, for studies requesting the deployment of back-mounted devices on penguins for long-term duration, we recommend avoiding the use of glue on feathers, and to use instead a mix of Tesa® tape strips (feathers’ side) and epoxy (on the strips covering the device) to reinforce adhesion. We could not show that this attachment will last on adult emperor penguins as long as for juveniles. An early departure from the field due to logistical constraints prevented us to deploy this new technique on fully moulted adult emperor penguin. Adult emperor penguins experience very harsh environmental conditions on the sea ice, especially at their breeding site, during winter with temperatures below −50°C and wind speeds above 150 km/h at Atka Bay [82]. The difference of habitats between adults and juveniles or other parameters (e.g. feathers’ properties) could make the back-taped-epoxied technique less efficient on adults than on juveniles. However, average deployment durations on adults are less than six months (Table 2) and need to be improved to cover the entire breeding cycle and justify the impacts on the birds’ welfare. We are convinced that new techniques should be tested such as the promising one presented in this study for juveniles.

#### Long-term deployment on adults: Leg-banded loggers

To collect year-round data (pressure and temperature) at a relatively high frequency (1 Hz), we developed and tested the first leg-band TDR mount for adult emperor penguins. These data will allow a detailed analysis of foraging activities and water column exploitation over a full year for the same birds. Although leg-band mounted devices had already been deployed on other penguin species [42–45, 83–85], the condition of the birds at retrieval are often not mentioned. However, some of the studies reported similar leg irritations as the ones we observed in this study ([42, 85]; Raclot personal communications; Houstin, Fournier and Le Bohec, unpublished observations). Such irritations might be due to the fact that emperor penguins can walk over long distances on sea ice to reach the water [74], not to mention that given the extremely cold habitats of emperor penguins, such leg-band might induce some chilling for the equipped bird. The commonly accepted flying bird banding technique is also known to cause unintentional damage like sores, inflammation, or even loss of feet in extreme cases [86–88], thus the irritations observed here can be considered as a minor impact.

We suggest that the use of non-continuous heat-shrink tubing and the glue around the head of the rubber cable tie created a small ledge in the otherwise smooth surface that irritated the birds’ leg-skin. From this observation, we have designed an improved version (S1 Slideshow in S1 File), which could not be tested due to early departure from the field site. The continuous heat-shrinkable sheath in the updated bracelet attachment will likely reduce friction between the leg and the bracelet and ideally avoid skin irritation. We expect the tibiotarsus to be less irritated and the occasional development of sores prevented. To prevent the formation of glue flakes, glue should be applied inside the cable tie’s closure and not around the whole head. At retrieval, the mounting did not show any damage or sign of wear and is expected to last several years before the elastomeric cable tie breaks.

As a result, with this bracelet technique, multi-year deployments might be considered. Scientific programs running in Antarctica are not always able to return several years in a row, and this technique of deployment offers some flexibility. Solutions still need to be developed for communicating devices (GPS, ARGOS) or for biologgers that would record too noisy data when positioned on the leg. However, for small loggers able to record environmental variables (*e*.*g*. hydrostatic pressure, water conductivity and temperature, luminosity) on a multi-year scale [81, 89], the leg-band technique appears as a promising potential alternative. Emperor penguins are non-nesting seabirds, breeding freely on sea ice within a mobile colony [90], making the recapture of birds difficult, especially after more than one year when the annual moult removed any externally painted-markings. However, thanks to their PIT-tag, all birds manipulated can be life-long identified by automatic Radio-frequency identification (RFID) detection systems [91–94]. Such systems have been successful at detecting emperor, Adélie and king (*Aptenodytes patagonicus*) penguins over the last decade at Pointe Géologie, Crozet and Kerguelen archipelagos (85; Le Bohec, Houstin, Chatelain and Courtecuisse, unpublished observations). By deploying such systems, the retrieval ratio of 50% of our technique will certainly be improved and reach similar retrieval rate than for nesting birds, *i*.*e*. between 60 and 90% [42, 43, 84, 85, 95, 96].

## Conclusion

Ethical concerns raised by the use of measuring devices on wild animal are not new [17] and a recent review [97] addressed the current pros and cons on attachment issues. To ensure exemplary data quality from a scientific and ethical perspective, the potential deleterious effects of deployment procedures (capture-attachment-recapture) must be evaluated and mitigated. Our study provides very detailed procedures to capture, recapture and externally attach biologgers to emperor penguins. We, therefore, consider this study as a significant advancement by (i) showing the impact of using glue for biologging device attachment on emperor penguins, (ii) helping to assess long-term loggers loss reasons (notably ARGOS transmitters), (iii) presenting two promising attachment techniques of biologging devices on emperor penguins in detail, and (iv) explicitly providing techniques to capture and handle emperor penguins with a limited amount of disturbance as well as a maximum of safety and efficiency. This publication is intended to serve as a resource to facilitate future research on this species.

## Supporting information

S1 File (TXT)
